# A Multiple Antibiotic-Resistant *Enterobacter cloacae* Strain Isolated from a Bioethanol Fermentation Facility

**DOI:** 10.1264/jsme2.ME13162

**Published:** 2014-06-17

**Authors:** Colin A. Murphree, Qing Li, E. Patrick Heist, Luke A. Moe

**Affiliations:** 1 Department of Plant & Soil Sciences, University of Kentucky, Lexington, KY, USA 40546–0312; 2 Ferm Solutions, Inc., 445 Roy Arnold Ave., Danville, KY, USA, 40422

**Keywords:** *Enterobacter cloacae*, bioethanol, fermentation, antibiotic resistance

## Abstract

An *Enterobacter cloacae* strain (*E. cloacae* F3S3) that was collected as part of a project to assess antibiotic resistance among bacteria isolated from bioethanol fermentation facilities demonstrated high levels of resistance to antibiotics added prophylactically to bioethanol fermentors. PCR assays revealed the presence of canonical genes encoding resistance to penicillin (*ampC*) and erythromycin (*ermG*). Assays measuring biofilm formation under antibiotic stress indicated that erythromycin induced biofilm formation in *E. cloacae* F3S3. Planktonic growth and biofilm formation were observed at a high ethanol content, indicating *E. cloacae* F3S3 can persist in a bioethanol fermentor under the highly variable environmental conditions found in fermentors.

The major increase in bioethanol production in the United States has brought to the forefront issues with large-scale fuel ethanol fermentations, most pressingly perhaps that with bacterial contamination of the fermentation apparatus. Contaminating bacteria can outcompete the yeast for the available nutrients, resulting in a process referred to as “ bacterial bloom”. During the bloom event, the contaminating bacteria consume the fermentable sugars, producing organic acids rather than ethanol. These events can result in significant financial losses for the producer through the loss of the fouled fermentation and the fermentable sugars in addition to cleaning and repairs. Facilities with repeated bloom events have used the prophylactic addition of antibiotics with some success to eliminate bacteria from the fermentation apparatus. Antibiotics used for this purpose include representatives of the β-lactam (*e.g.*, penicillin), macrolide (*e.g.*, erythromycin), and streptogramin (*e.g.*, virginiamycin) classes. While members of the Gram positive lactic acid bacteria (LAB) clade are the most common bacterial inhabitants of the bioethanol fermentation apparatus, certain Gram negative bacteria have been cultured from these facilities as well (5, 15). Concerns have arisen regarding antibiotic resistance among bacteria in bioethanol fermentors, and antibiotic resistance among certain LAB has been noted previously (4, 13). Among the Gram negative bacteria isolated from bioethanol facilities, however, instances of antibiotic resistance have yet to be reported until now.

Here we describe an *Enterobacter cloacae* strain isolated from a bioethanol facility that exhibited resistance to each of the antibiotics commonly used in the bioethanol industry. Similar to other members of the family *Enterobacteriaceae*, *E. cloacae* exhibits a fairly cosmopolitan distribution. It is a common inhabitant of the human microbiome, in which it is routinely found in the gastrointestinal tract (8). *E. cloacae* has also been detected in soil and associates with plants, in which it can be pathogenic or beneficial (10, 16, 17). It remains unclear whether members of the family *Enterobacteriaceae* found in bioethanol fermentations originate from plant material or from humans. While LAB are commonly considered the most problematic bacteria for bioethanol producers, the potential for bloom due to Gram negative bacteria is currently unknown.

The *E. cloacae* isolate was cultured from a corn mash-based bioethanol fermentation facility (Murphree *et al.*, kept confidential for the purpose of publication). This was part of a larger project in which bioethanol mash samples from a number of facilities across the US were assessed for culturable, antibiotic-resistant bacteria. Bacteria were isolated by culturing the sample on MRS medium supplemented with 0.5 ppm of penicillin, erythromycin, and virginiamycin. Genomic DNA was purified from the liquid culture using the GenElute Bacterial Genomic DNA Kit (Sigma-Aldrich, St. Louis, MO). The partial 16S rRNA gene was amplified from genomic DNA using the 27F and 1492R primers (Table 1) (11). The cloned 16S rRNA gene sequence was queried against GenBank using a nucleotide BLAST (2). Of greater than 300 bacterial strains identified in multiple bioethanol fermentation facilities across the US, this strain was the only Gram negative, multiple antibiotic-resistant isolate. The 16S rRNA gene sequence revealed that this bacterium was closely related to members of the *E. cloacae* complex (99% identity to *E. cloacae* subsp. *dissolvens* strain SB 3013, GenBank accession no. GU191924.1), and the strain was named according to the facility (facility 3) and strain number (strain 3) from that facility (*Enterobacter cloacae* F3S3).

Minimal inhibitory concentration (MIC) assays were conducted in MRS medium in sterile, flat-bottom 96-well plates according to the Clinical and Laboratory Standards Institute (CLSI) standards (1). Penicillin (Research Products International [RPI], Mt. Prospect, IL), erythromycin (RPI), or FermGuard Sentry (Ferm Solutions, Danville, KY) were used independently in MIC assays. FermGuard Sentry is a formulation of virginiamycin that is used in bioethanol fermentations. The FermGuard Sentry was designated with 50% activity; thus, wt vol^−1^ measurements were doubled to account for the inert ingredients in the virginiamycin preparation.

To determine whether *E. cloacae* F3S3 used an antibiotic inactivation mechanism to resist the activity of each antibiotic, a zone-of-inhibition assay was used. An MRS agar plate was seeded with a susceptible bacterial strain (*Leuconostoc pseudomesenteroides*) and three plugs were removed from the agar using the wide end of a sterile 200 μL pipette tip. The well was filled with 75 μL of filter-sterilized supernatant from an overnight culture of *E. cloacae* F3S3 in MRS broth supplemented with the antibiotic being assayed. Negative control plates used the same MRS broth without bacterial inoculation. Plates were incubated at 28°C for 24 h, and assays were classified as inactivating (yes) or non-inactivating (no) based on the radius of the zone of growth inhibition around the well (Table 2). Assays in which the zone of inhibition was less than or equal to half the radius of the negative control plate were considered to be inactivating. PCR assays were performed to detect canonical resistance genes for β-lactams: *ampC*; macrolides: *ere*(A, B), *erm*(A, B, C, G), *erm*(G, T), *mef*(A, E), *mph*(A, B, C), *mph*(D), *mph*(E), *mph*(F), *msr*(A, B); and streptogramins: *lsa*(A, C), *vat*(A, C, F), *vat*(B, D, E), *vat*(H), *vga*(A), *vga*(B), *vgb*(A), and *vgb*(B). The sequences for PCR primers and cycling conditions are listed in Table 1. Regarding PCR primers not previously used for this purpose, consensus primers were designed based on one or more gene sequences per gene class (Table 1). PCRs showing an appropriately sized product by gel electrophoresis were purified, and their products were then cloned into pGEM-T. The DNA insert was sequenced and the cloned DNA was queried against GenBank to identify the (partial) gene sequence and also to determine to which resistance gene class the sequence belonged.

MIC data in Table 2 show that *E. cloacae* F3S3 exhibits elevated levels of resistance to each of the three types of antibiotics that are commonly applied prophylactically in bioethanol fermentation. Canonical antibiotic resistance genes were amplified for the β-lactamase *ampC* and the erythromycin ribosomal methyltransferase *ermG*. Despite assaying for multiple virginiamycin resistance gene classes (Table 1), no canonical genes were amplified. This suggests that either the strain uses a heretofore unidentified mechanism for resistance, or that the PCR primers are not optimized to account for divergent sequences within these gene classes. Table 2 also shows that *E. cloacae* F3S3 erythromycin resistance is not mediated by antibiotic inactivation, as opposed to resistances to penicillin and virginiamycin. This is consistent with the mode of action of both the *ampC*-encoded β-lactamase and the *ermG*-encoded ribosomal methyltransferase.

Biofilm formation assays were performed in sterile, flat-bottom 96-well plates using MRS medium according to the crystal violet stain assay of Stepanovic *et al.* (19). Wells containing MRS medium without bacterial inoculation were used as a negative control. The mean OD_570_ of the assay wells and the negative control wells (OD_c_) were used to assess biofilm formation. As per Stepanovic *et al.*, an OD_570_ greater than the OD_c_ indicated that the strain was a biofilm former (19). Planktonic growth (OD_600_) was measured in a new 96-well plate on the liquid culture removed from each plate assay. To assess the impact that sub-MIC concentrations of antibiotics or ethanol have on *E. cloacae* F3S3 biofilm formation, penicillin, erythromycin, or virginiamycin were added at a final concentration of 0.5 μg mL^−1^ to the assay well at inoculation, or ethanol was added to a final wt vol^−1^ concentration of either 3% or 7% to the assay well.

Fig. 1 shows the results obtained from a combined biofilm/planktonic growth assay. According to the biofilm assay parlance of Stepanovic *et al.* (19), *E. cloacae* F3S3 was a “weak” biofilm producer (OD_C_<OD_570_<2×OD_c_), which is generally consistent with what is known about *Enterobacter* isolates (9). Fig. 1 also shows that the sub-MIC levels of either penicillin or virginiamycin had negligible effects on biofilm formation by *E. cloacae* F3S3 (which may be expected based on antibiotic inactivation), whereas erythromycin at 0.5 μg mL^−1^ significantly induced biofilm formation (*p*=0.036).

With increasing ethanol content, planktonic growth clearly decreases, but growth is still seen at 7% wt vol^−1^ (Fig. 1) indicating that the strain can persist under ethanol conditions seen during fermentation. Biofilm formation was also noted, but the levels of biofilm formation decreased as ethanol concentration increased, indicating that biofilm formation was not induced by increased ethanol concentrations.

While antibiotic resistance among the *E. cloacae* complex has been reported previously among human isolates, the broader ecology of antibiotic resistance among members of this species—especially among environmental isolates—has not yet been fully clarified. Here we demonstrated that *E. cloacae* F3S3 could persist under conditions that were not typically amenable to bacterial growth. This strain exhibited high levels of resistance to β-lactams, macrolides, and streptogramins. It also grew in elevated ethanol levels, and could form biofilms under conditions similar to those that occur during fermentation. These results should draw attention to the potential of Gram negative bacteria to disrupt bioethanol fermentations, and further work should address the ecology of antibiotic resistance among those members of the *E. cloacae* complex not isolated from human sources.

The partial gene sequences resulting from this work have been submitted to GenBank under accession numbers KF562730 (16S rRNA gene), KF672185 (*ampC* gene), and KF562731 (*ermG* gene).

## Figures and Tables

**Fig. 1:**
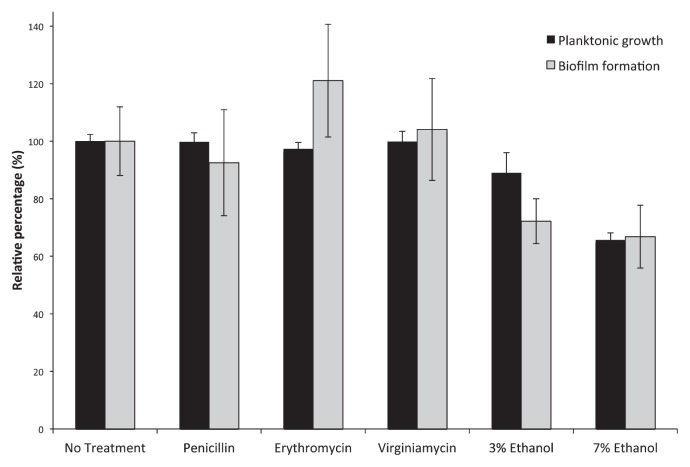
Planktonic growth and biofilm formation by *E. cloacae* F3S3 in MRS medium (No Treatment), MRS medium supplemented with sub-MIC concentrations of penicillin, erythromycin, or virginiamycin (0.5 μg mL^−1^), or with ethanol at 3% or 7% wt vol^−1^. “No treatment” values for planktonic growth (OD_600_ values) and biofilm formation (OD_570_ values) were normalized to 100% to enable direct comparison between treatments. Biofilm formation was observed under all growth conditions (OD_570_>OD_c_). Pairwise *t*-tests revealed statistically significant (0.05 or less) *p*-values in planktonic growth (3% ethanol, *p*=0.0026; 7% ethanol, *p*=8.9E-14) and biofilm formation (erythromycin, *p*=0.036; 3% ethanol, *p*=0.00037; 7% ethanol, *p*=0.00010) compared to the untreated control.

**Table 1:** PCR primers used in this study^a^,^b^

Target genes or primer name		Primer sequence (5′-3′)	Anneal temp.	GenBank entries used for the primer design *or* reference for the primer origin
*ampC*	F:	GACAAAATCCCTTTGCTG	50°C	NC_018405.1
	R:	CTCAGAATACGGTATGC		
*ere*(A, B)	F:	CTCATTTYRTMRMRGARTT	45°C	AY183453, A15097
	R:	GGWGTTTTTTGWAKATG		
*erm*(G, T)	F:	AAATATAAAAGATAGTCAAAA	45°C	L42817.1, M64090.1
	R:	CCATATTCCACTATTAAATAAG		
*mph*(A, B, C)	F:	TGGGTKCTRMGMWTSCCK	50°C	D16251, D85892, AB013298
	R:	ARCCCYTCTTCMCCAAA		
*mph*(D)	F:	CTCCTGTAACCAAGCCAATTG	55°C	AB048591
	R:	TTATCAACCCCGACCAGATTA		
*mph*(E)	F:	ATGACAATTCAAGATATTCAATC	50°C	FR751518
	R:	TTATATAACTCCCAACTGAGC		
*mph*(F)	F:	ATGCTGCACGACACGGACCG	55°C	AM260957
	R:	TCAAATCCCTGGCGCCGAC		
*vat*(A, C, F)	F:	ATTGGDGATAARYTRAT	45°C	L07778, AF015628, AF170730
	R:	ACMGGCATAATBRWYACATC		
*vat*(B, D, E)	F:	TTATYATGAAYGGWGCMAAYCA	50°C	U19459, L12033, AF139725
	R:	ATKGCWCCRTCHCCKATTT		
*vat*(H)	F:	ATGGCAGAAAAATTAAAAGG	45°C	GQ205627.2
	R:	CTAATCATTTTCTTTAGAAA		
*vgb*(B)	F:	GTTTCTATGCTGATCTGAATC	50°C	AF015628
	R:	GGTCTAAATGGCGATATATATGG		
*mef*(A, E)	F:	AGTATCATTAATCACTAGTGC	50°C	(20)
	R:	TTCTTCTGGTACTAAAAGTGG		
*vga*(A)	F:	CCAGAACTGCTATTAGCAGATGAA	55°C	(6)
	R:	AAGTTCGTTTCTCTTTTCGACG		
*vga*(B)	F:	TGACAATATGAGTGGTGGTG	55°C	(6)
	R:	GCGACCATGAAATTGCTCTC		
*vgb*(A)	F:	ACTAACCAAGATACAGGACC	50°C	(12)
	R:	TTATTGCTTGTCAGCCTTCC		
*lsa*(A, C)	F:	GGCAATCGCTTGTGTTTTAGCG	55°C	(18)
	R:	GTGAATCCCATGATGTTGATACC		
*erm*(A, B, C, G)	F:	GAAATIGGIIIIGGIAAAGGICA	37°C	(6)
	R:	AATTGATTCTTIGTAAA		
*msr*(A, B)	F:	GCAAATGGTGTAGGTAAGACAACT	55°C	(6)
	R:	ATCATGTGATGTAAACAAAAT		
27F		AGAGTTTGATCCTGGCTCAG		(11)
1492R		GGTTACCTTGTTACGACTT		(11)
337F		GACTCCTACGGGAGGCWGCAG		(7)
785F		GGATTAGATACCCTGGTA		(7)
M13	F:	GTTTTCCCAGTCACGAC		
	R:	CAGGAAACAGCTATGAC		

a 16S rRNA gene PCRs used 20 ng genomic DNA, primers at a final concentration of 0.5 μmol L^−1^, and the DreamTaq DNA Polymerase Master Mix (Fermentas, Glen Burnie, MD). Cycling parameters were: 95°C for 2 min, and 30 cycles of 95°C for 30 s, 55°C for 30 s, 72°C for 2 min, with a final extension at 72°C for 30 min. The PCR product was cloned into pGEM-T (Promega, Madison, WI) and sequenced using the PCR primers M13F and M13R. The PCR primers 337F and 785F were used to obtain complete coverage of the cloned 16S rRNA gene (7).

b Antibiotic-resistant gene PCRs comprised 1 ng μL^−1^ genomic DNA, 0.5 μmol L^−1^ of each primer, and the DreamTaq DNA Polymerase Master Mix (Fermentas). PCRs used initial denaturation (10 min at 95°C), followed by 30 cycles of denaturation (30 s at 95°C), annealing (30 s, temperatures indicated above), and elongation (2 min at 72°C), followed by a final elongation step of 30 min at 72°C. PCRs using the *erm* (A, B, C, G) primer set used the parameters from Arthur *et al.* (3). PCR primers are further described in Murphree, *et al.* (14).

**Table 2:** *E. cloacae* F3S3 antibiotic resistance

Antibiotic	MIC (μg mL^−1^)^a^	Inact.^b^	Gene^c^
Penicillin	8	Yes	*ampC*
Erythromycin	64	No	*ermG*
Virginiamycin	>512	Yes	NA^d^

a Minimum inhibitory concentration

b Antibiotic inactivation as determined by zone-of-inhibition assays

c Gene responsible for the antibiotic resistance phenotype

d No canonical virginiamycin resistance gene was identified
